# Comparative synthesis of tandem repeats in the control region of *Epinephelus* mitogenomes (Peciformes: Epinephelidae)

**DOI:** 10.1590/1678-4685-GMB-2025-0240

**Published:** 2026-05-15

**Authors:** Carla Bessa-Brito, Rodrigo Petry Corrêa de Sousa, Wanny Gomes de Lima, Renata Furtado do Rosário, Luan Pinto Rabelo, Yrlan Sousa Oliveira, Iracilda Sampaio, Grazielle Fernanda Evangelista Gomes, Marcelo Vallinoto

**Affiliations:** 1 Universidade Federal do Pará, Instituto de Estudos Costeiros, Laboratório de Evolução, Bragança, PA, Brazil.; 2 Instituto Tecnológico Vale (ITV), Belém, PA, Brazil.; 3 Universidade Federal do Pará, Instituto de Estudos Costeiros (IECOS), Laboratório de Genética Aplicada (LAGA), Bragança, PA, Brazil.; 4 Universidade do Porto, Centro de Investigação em Biodiversidade e Recursos Genéticos, Vairão, Portugal.

**Keywords:** Mitogenomes, VNTR, concerted evolution, phylogeny

## Abstract

In this study, we analyzed the evolutionary patterns of tandem repeats in the mitochondrial Control Region (CR) of groupers of the genus *Epinephelus*. This research was based on a comprehensive survey of *Epinephelus* mitogenomes available in public databases, aiming to recover complete CR sequences. A total of 97 specimens were recovered, including 31 species that contain tandem repeat sequences. The repeated sequences were classified into three categories according to their length: short, medium, and long. The long repeats were restricted to a monophyletic group, while short and medium repeats were more widely distributed across different clades, whereas some species lacked repeats entirely. Given the similarities found among the repeat sequence alignments and the phylogenetic arrangements, it was possible to infer that these segments evolved in synchrony over time, with a process of concerted evolution being the most likely explanation for the evolutionary dynamics of these repetitive DNAs. Correlation analyses further revealed that mitogenome length covaries with CR length and that CR length increases with the full repeat region length, whereas motif length is inversely related to repeat copy number. These patterns underscore how repeat architecture evolves through time in the mitochondrial CR of epinephelid mitogenomes.

## Introduction

The groupers of the family Epinephelidae are reef-dwelling marine fish that include 249 species distributed in 32 genera (Fricke *et al.,* 2025). The species of this family are highly valued as ornamental fish, particularly as top-quality seafood (Sadovy *et al.*, 2013). *Epinephelus* is the most diverse genus of Epinephelidae, with more than 90 recognized species (Nelson et al., 2016; Ma and Craig, 2018; Frable et al., 2019). Many of these species, such as *Epinephelus itajara* and *Epinephelus akaara*, are listed as either vulnerable or endangered as a result of population decline caused by overfishing and the degradation of habitats (Silva-Oliveira et al., 2008; Sadovy *et al.*, 2013; Bertoncini et al., 2018).

Given the importance of these fishes, several studies have used sequences of mitochondrial DNA (mtDNA) to resolve phylogenetic, phylogeographic or population-level questions, with a conservation focus (Pinhal et al., 2012; Sodré et al., 2012; Guimarães-Costa et al., 2016; Cárcamo-Tejer et al., 2021; Sun et al., 2021; Davidović et al., 2022). The mtDNA has been an important tool for determining indices of genetic diversity in the grouper, making some important contributions to studies in both evolution and conservation, as well as providing molecular tools for the identification of species (Damasceno et al., 2015; Qu et al., 2018; Fadli et al., 2021), metabarcoding (Li et al., 2023; Qing et al., 2024) or phylogenomics (Song et al., 2024; Winn et al., 2024).

Notably, the mtDNA is a potentially important marker for molecular studies because of its unique set of characteristics, such as haploidy, easy extraction, and, in particular, its high mutation rates (Lawless et al., 2020). The arrangement of this molecule is also highly conserved in vertebrates, with approximately 16-17 kb, no introns and only 37 genes (13 encoding proteins, two for rDNA, and 22 tRNAs), in addition to a noncoding region, known as the Control Region (CR) (Zhuang et al., 2013). 

The mitochondrial CR plays a central role in regulating mitochondrial DNA replication and transcription, and it is commonly described as comprising three domains (Fonseca et al., 2014; Bernacki and Kilpatrick, 2020). The 5’ domain contains termination-associated sequences of the heavy strand, in addition to a varying number of tandem repeats. The central domain contains conserved sequence blocks (CSBs), whereas the 3’ domain typically includes three short conserved sequence blocks (CSB-1, CSB-2, and CSB-3). These elements are associated with the initiation of H-strand replication and with transcriptional regulation via the light-strand promoter (LSP) and heavy-strand promoter (HSP) (Clayton, 1992), and the 3’ domain may also harbor tandem repeats. This interesting feature may involve considerable variation in the number of tandem repeats or VNTR (Morris-Pocock et al., 2010; Akiyama et al., 2017; Shu et al., 2018; Kornienko et al., 2019). These repeats typically contain between 10 and 100 base pairs (bps) and thus have a considerable influence on some aspects of the mitochondrial genome, including its size, structure, replication, and recombination (Røyrvik and Johnston, 2020; Lin et al., 2021; Thapana et al., 2022).

Several studies have shown that VNTR configurations are often similar among closely related species and even among populations within the same species, reflecting concerted evolution that homogenizes repeats within a genome (Feliner and Rosselló, 2012; Gomes et al., 2016; Belyayev et al., 2019). This mechanism is a biological process that results from the recombination of the DNA through the unequal exchange of the units of gene repetition, repair, and conversion, ultimately resulting in a similar set of repetitive sequences (Calonje et al., 2009; Gomes *et al*., 2016; Akiyama et al., 2017). 

Recent mitogenomic sequencing efforts in the genus *Epinephelus* have identified numerous tandem repeats, although these studies have focused primarily on genome characterization and CR description (Han J et al., 2011; Han S *et al.*, 2014; Cheng et al., 2015; He et al., 2024; Padgett and Baeza, 2025). Given these limitations, the application of an evolutionary perspective to the investigation of the origin, structure, and dynamics of these segments may provide important insights into the genomic diversity and evolution of these repeats in the genus *Epinephelus*. For example, what is the relationship between the different types of repeats in phylogenetically closely related species? Do these phylogenetic relationships reveal how the repetition motif expanded? Additionally, is it possible to infer when a repetition motif increased in size? In this context, this review examines the evolutionary trends of tandem repeats in the mitochondrial CR of *Epinephelus* species, exploring their importance for comprehending phylogenetic connections and the dynamics of repeat size fluctuations.

## Material and Methods

### Data acquisition and database compilation

Complete mitochondrial genomes from the Epinephelidae family, of the genus *Epinephelus*, were obtained through a comprehensive search of the GenBank public online DNA sequence database, which is available on the platform of the National Center for Biotechnology Information (NCBI). Only complete and annotated mitogenomes were analyzed, and no raw sequences were used. Thus, all analyses used assembled genomes from public repositories.

The search strategy was based on the current taxonomic structure proposed by Ma and Craig (2018). The accession numbers, species names, and taxonomic information are provided in Table 1 and Table S1. Species from genera other than *Epinephelus* were included for comparative purposes and were also used as outgroups in phylogenetic analyses.

**Table 1 -:** Results of the analyses of the genus *Epinephelus* run using Tandem Repeats Finder. The asterisks (*) indicate the species that have more than one repeat. The numbers highlighted in bold represent the largest tandem repeats identified within each species, evidencing intraspecific variability in repeat length, which is further characterized in detail in Table S1.

Species	Number of genomes analyzed	Size of the genome (bps)	Size of the control region (bps)	Full Repeat Region Length	Motif Length (bps)	Type	Number of Repeat Units	VNTR
*Epinephelus lanceolatus*	5	16714 - 16743	880 - 1041	1 - 134	17	Short	7.8 - 17.8	Yes
*Epinephelus fuscoguttatus*	3	16373 - 16648	673 - 948	1 - 190	17	Short	10.9	No
*Epinephelus coioides*	3	16418 - 16458	720 - 760	1 - 153	20	Short	5.8 - 7.8	Yes
*Epinephelus trimaculatus*	3	16761 - 16777	1056 - 1074	166 - 366	18	Short	10.2 - 11.2	Yes
*Epinephelus moara*	3	16696	998 - 999	1 - 373	17	Short	22.1	No
*Epinephelus bruneus*	3	16686 - 16692	991 - 995	1 - 372	17	Short	22 - 22.1	Yes
*Epinephelus epistictus*	2	16920	1217	7 - 553	33	Short	16.4	No
*Epinephelus corallicola*	2	16647	950	101 - 249	18	Short	8.3	No
*Epinephelus malabaricus*	3	16423	720	1 - 113	20	Short	5.8	No
*Epinephelus tukula*	2	16503	805	8 - 170	21	Short	7.7	No
*Epinephelus septemfasciatus*	2	16558	850	10 - 230	18	Short	12.3	No
*Epinephelus tauvina*	2	16787	1080	214 - 307	22	Short	4.2	No
*Epinephelus quoyanus*	2	16797	1093	181 - 382	18 or 36	Short	11.2 or 5.6	No
*Epinephelus bontoides*	2	16903	1200	173 - 497	37	Short	8.8	No
Epinephelus chlorostigma *	2	16894	1191	505 - 556	23	Short	2.3	No
Epinephelus chlorostigma *				52 - 503	76	Medium	5.9	
*Epinephelus areolatus*	3	16893 - 16965	1191 - 1264	52 - 579	76	Medium	5.9 - 6.7	Yes
*Epinephelus akaara*	5	16795	1093	20 - 435	133	Long	3.1	No
*Epinephelus awoara*	3	16798 - 16802	1098	21 - 427	134	Long	3 - 3.2	Yes
*Epinephelus fasciatomaculosus*	2	16682	980	6 - 349	141	Long	2.4	No
*Epinephelus sexfasciatus*	2	16786	1090	14 - 422	132	Long	3.1	No
*Anyperodon leucogrammicus*	1	16616	916	3 - 254	17	Short	14.8	No
*Epinephelus amblycephalus*	2	16869	1169	47 - 419	50	Medium	7.5	No
*Epinephelus adscensionis*	2	16963	881	34 - 227	19	Short	10.2	No
*Epinephelus hexagonatus*	2	16872	1172	197 - 447	17	Short	14.8	No
*Epinephelus itajara*	1	16561	857	1 - 205	17	Short	12.1	No
*Epinephelus marginatus*	1	16984	1286	46 - 541	57	Medium	8.7	No
*Epinephelus morio*	2	16756	1057	1 - 462	18	Short	25.7	No
*Epinephelus polyphekadion*	2	16619	915	27 - 140	18	Short	6.3	No
*Epinephelus undulosus*	2	17126	954	29 - 189	10	Short	16.1	No
Epinephelus bilobatus*	1	17354	981	169 - 209	2	Short	20.5	No
Epinephelus bilobatus*				838 - 874	8		4.6 - 4.8	Yes
*Epinephelus maculatus*	3	17066 - 16746	924 - 937	726 - 785	29	Short	2.1	No
*Epinephelus merra*	2	17017	988	-	-	-	-	-
*Epinephelus bleekeri*	2	17227	900	-	-	-	-	-
*Epinephelus latifasciatus*	2	16389	684	-	-	-	-	-
*Epinephelus stictus*	2	16524	824	-	-	-	-	-
*Epinephelus aeneus*	1	16578	891	-	-	-	-	-
*Epinephelus multinotatus*	2	17057	766	-	-	-	-	-
*Epinephelus cyanopodus*	2	16649	768	-	-	-	-	-
*Epinephelus flavocaeruleus*	2	16758	767	-	-	-	-	-
*Epinephelus japonicus*	1	17084	1091	-	-	-	-	-
*Epinephelus longispinis*	1	17221	954	-	-	-	-	-
*Epinephelus altivelis*	4	16503 - 16497	799 - 805	-	-	-	-	-
*Hyporthodus haifensis*	1	16525	798	-	-	-	-	-
*Hyporthodus octofasciatus*	2	16545	840	-	-	-	-	-

The asterisks (*) indicate the species that have more than one repeat. The numbers highlighted in bold represent the largest tandem repeats identified within each species, evidencing intraspecific variability in repeat length, which is further characterized in detail in Table S1.

### Extraction and characterization of the mitochondrial control region

The mitochondrial CR was identified on the basis of existing genomic annotations available in GenBank. When necessary, CR boundaries were manually refined by locating the region between the tRNA-Pro and tRNA-Phe genes, following the canonical organization of vertebrate mitogenomes. The total mitogenome length and CR length were recorded, and only CR sequences derived from complete mitochondrial genomes were included in subsequent analyses.

### Identification of tandem repeats

Tandem repeats within the mitochondrial CR were identified via Tandem Repeats Finder (TRF) version 4.07b (Benson, 1999). Analyses were performed via the software’s default settings, which enable detection of a set of repeat descriptors, such as the consensus motif sequence, including its nucleotide composition (the Motif Length of the repeat unit that is copied in tandem within the array; that is, the consensus repeat unit reported by TRF), and Repeat Copy Number (the number of repeat units, that is, how many times the motif is consecutively repeated within the array) and the Full Repeat Region Length (start-end of the repeat) within the CR. 

When more than one repeat array was detected in the same CR, each array was treated as an independent record. For descriptive purposes, the motifs were grouped into three practical classes the basis of motif length: short, medium, and long. The medium category was used for intermediate cases that did not fit naturally into the short or long classes.

All repeat arrays detected by TRF are summarized in Table 1 and Table S1. To compare repeats within and between species, we focused on the motif itself. The consensus motif sequences reported by TRF, that can be found in the supplementary material (Table S1), were aligned via MAFFT v7.313, and similarity was assessed via direct comparison of the aligned motifs, together with motif length and repeat copy number. Intraspecific comparisons were performed when more than one specimen was available for a species, and variation was recorded primarily as differences in repeat copy number. We refer to VNTR-like variation when specimens of the same species share the same or highly similar motif sequence but differ in the number of repeat units. Interspecific comparisons were based on motif sequence similarity across taxa, allowing us to identify shared motif types and to evaluate how these patterns were distributed across the phylogenetic framework.

### Phylogenetic analyses 

Phylogenetic analyses were conducted on the basis of the sequences of 13 protein-coding genes from 41 species of *Epinephelus*, along with three species of the genus *Hyporthodus*. Additionally, *Cephalopholis sonnerati*, *Variola louti*, *Plectropomus areolatus* and *Grammistes sexlineatus* were included as outgroup taxa (Table 1; Table S1). The sequences were aligned via MAFFT v7.313 software (Katoh and Standley, 2013) and then concatenated via SequenceMatrix v1.10 (Vaidya et al., 2011). The database was used for the production of a maximum likelihood tree in IQTREE v3.0.1 software (Nguyen et al., 2015), and ultrafast bootstrap analysis, with 1,000 pseudoreplicates, was used to determine the statistical support for each node. The IQTREE automatic substitution model options were used to select the evolutionary model for each partition.

### Statistical analyses

Statistical analyses were performed to evaluate the contribution of tandem repeats to length variation in mitochondrial genomes, with a focus on the CR. We applied both Pearson’s (*r*) and Spearman’s rank (*ρ*) correlation tests to examine a specific set of structural hypotheses. The choice of these methods followed normality assessments via the Shapiro-Wilk test. While mitogenome and CR lengths followed a normal distribution (*p > 0.05*), repeat-related variables (motif length, repeat copy number and full repeat region length) deviated significantly (*p < 0.05*). Consequently, Spearman’s *ρ* was prioritized as a robust estimator for nonparametric associations, whereas Pearson’s *r* provided a measure of linear expansion.

First, we tested whether the overall mitogenome length covaries with CR length. Because repeat arrays can vary through two components, motif length and copy number, we evaluated whether CR length or mitogenome length are better explained by the full repeat region length. Finally, because this region occupies a limited segment of the CR, we tested whether longer motifs tend to occur in fewer copies or shorter motifs tend to occur in more copies. 

## Results

**Figure 1 -:**
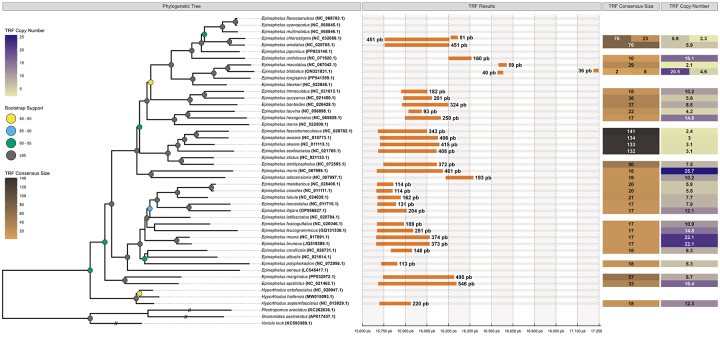
Phylogeny of groupers based on 13 protein-coding genes. The TRF Consensus Size panel indicates the lengths of the tandem repeat cores, with different colors representing short, medium, and long repeat motifs. The TRF Number of Copies panel shows the number of times the core is repeated. The TRF Results panel displays the distribution of repeats along the control region, predominantly in the 5’ domain, and their correspondence within the phylogeny.

The CR sequences were selected from the complete genomes of 97 specimens belonging to seven genera (Table S1). Tandem repeats were detected in 31 species (Table 1), and the alignment of the sequences revealed that the repeats were concentrated primarily in the 5’ domain. In 13 of the species that had repeats, these motifs began between bases 1 and 10 of the CR, including 9 species in which they began between bases 1 and 3 (Table 1 and Figure 1).

Many sequences are unique to a given taxon, which precludes the conclusive confirmation of the existence of VNTRs for all species. However, in the species for which at least two sequences were available, only seven had VNTRs (Table 1). The motifs of the repetitive sequences vary in size from 17-141 bps, which permitted the classification of the repeats into three categories: short (2-37 bps), medium (57-76 bps), and long (132-141 bps) (Table 1). Some of the repeated sequences are highly homogeneous in terms of motif sequence composition, such that both their alignments and distribution in the phylogeny confirm the similarities between the repetitive sequences (Figure 1). 

Two unique species, E*pinephelus chlorostigma* (KR872887.1, and NC_032086.1) and *Epinephelus bilobatus* (ON321831.1 and NC_069198.1), presented two types of repeats. In *E. chlorostigma,* one medium repeat of 76 bps followed by one short repeat of 23 bps were identified; these repeats were arranged *in tandem* and located between bases 52-503 and 505-556, respectively. In *E. bilobatus,* two short repeats were found, positioned between bases 169-209 and 838-879 (Table 1; Figure 1).

In the phylogenetic analysis, long repeats were found exclusively in a single monophyletic group of species. In contrast, medium and short repeats were broadly distributed across the phylogeny, occurring in multiple clades and even in distinct genera (Figure 1). As mentioned previously, all the repetitions exhibited phylogenetic congruence. The alignment of the motif sequences can be easily performed, and these are well conserved among closely related species. For example, *Epinephelus tukula* (NC_024039.1 and KJ414470.1), *E. coioides* (NC_011111.1, EU043376.1, KM377093.1 and MW752082.1), and *E. malabaricus* (NC_028406.1 and KM873711.1) presented highly similar repeat sequences, reinforcing their close evolutionary relationships (Figure 1). Similarly, *E. bruneus* (NC_013820.1, JQ518289.1 and FJ594964.1) and *E. moara* (JQ518290.1, KP009977.1 and NC_017891.1) shared an identical repeat sequence, as did *Epinephelus itajara* (OP056827.1) and *Epinephelus lanceolatus* (NC_011715.1, OP980559.1, FJ472837.1, HQ660062.1, KM386619.1 and KJ451389.1), further supporting their recognition as sister species. In contrast, *E. tauvina* (NC_056898.1 and MW194890.1) and a set of closely related congeners presented a greater degree of motif sequence divergence, with sequence similarity decreasing in parallel with increasing phylogenetic distance.

In some species, the TRF identified more than one motif length for the same repeat sequence in the same region. For example, analysis of repeats in *Epinephelus quoyanus* revealed that the motif sequence occurs in two forms, differing in size, with repeats of 18 and 36 bps detected in the same region, between bases 181 and 382 (Table 1). The closely related species *E. trimaculatus* (NC_021612.1, KC847086. 1, and KC593372.1) and *E. bontoides* (NC_028428.1 and KT619054.1) also contained short repeats, with 18 bps in *E. trimaculatus* and 39 bps in *E. bontoides*. In other words, TRF was unable to distinguish the actual length of the motif, resulting in alternative computational interpretations that suggest different sizes for the same repeating motif sequence.

Finally, to test hypotheses linking tandem repeats to variations in the mitogenome and mitochondrial CR length, we performed correlation analyses. The results are summarized in Table S2 and all tests are supported by either Spearman’s rank or Pearson’s correlation analyses. First, CR length showed a strong positive association with overall mitogenome length (Test 1). Second, mitogenome length also increased with increasing length of the full repeat region (Test 2), indicating that repeat-rich segments can contribute to broader mitogenome length variation. Third, and most directly relevant to our main question, the mitochondrial CR length increased with the length of the full repeat region (Test 3), which is consistent with the idea that the physical footprint of the array translates into measurable differences in CR length.

In addition, motif length was strongly and inversely associated with the repeat copy number (Test 4). In practical terms, longer motifs tend to occur in fewer copies, whereas shorter motifs can be maintained in more copies, which is consistent with a limited segment of the CR being filled by different motif architectures over evolutionary time.

## Discussion

The present study revealed that Epinephelidae groupers share highly similar tandem repeat motif sequences both within species and among species. Belyayev et al. (2019) proposed that family-level satDNA mutations may initially spread gradually through mechanisms of homogenization to eventually become fixed in the population, resulting in the concerted evolution of these segments, a process observed in many different vertebrates (Lorite et al., 2017; Utsunomia et al., 2017; de Lima et al., 2020; Jiang et al., 2024; Dos Santos et al., 2024).

Other mechanisms, such as species hybridization, could also explain the similarities in repetitive sequences among some species. For example, alleles from one species can introgress into the gene pool of a second species through hybrids and, in some cases, the complete mitochondrial genome of another species can be incorporated into that of the first species (Harrison and Larson, 2014; Read et al., 2025). Hybridization events such as these are very common in the groupers (Kiriyakit et al., 2011; Gao et al., 2017; Murata et al., 2017; Chen et al., 2018; Triastuti et al., 2018; Shapawi et al., 2019; Fan et al., 2020; Li et al., 2020a ; Zhang et al., 2024; Aoki et al., 2025).

Nevertheless, hybridization alone cannot explain the full evolutionary history of the tandem repeats observed across *Epinephelus*, because it is limited to a subset of species known to hybridize; even in those cases, shared repeats would be expected only when introgression involves the same repeat type. However, although the species *E. tukula* and *E. moara* can hybridize naturally (Li et al., 2020 b ), they present different mitogenomes, as well as different repeats (Table 1; Table S1). In this case, the maintenance of the identity of these different groups of tandem repeats (Figure 1 and Table 1) could be better explained by the effects of concerted evolution on the CRs of these organisms, which are common observed both in this region in other species and multigene families (Hänske et al., 2020; Shi et al., 2020; Yano et al., 2020; Thapana et al., 2022; Jiang et al., 2024).

These tandem repeats result from a series of varied and complex mechanisms, two of which are cited most often in published studies: gene conversion linked to slippage and the concerted evolution of regions of the mitochondrial CR (Morris-Pocock et al., 2010; Akiyama et al., 2017; Lorite et al., 2017; Xiao et al., 2017; Wynn and Christensen, 2019; Jiang et al., 2024; Pham et al., 2024). Point mutation is another plausible mechanism. Following the appearance of this type of variation (i.e. base insertion or deletion), the processes mentioned above are fundamental to the variation in the size (increase or decrease) of the motif sequences of the repeats (Xiao *et al.*, 2017; Wynn and Christensen, 2019; Lin et al., 2021).

### Maintenance *vs*. elimination of repeats in Epinephelidae.

The groups of repeats identified in the groupers suggest an ancient origin, with an evolutionary dynamic that is best understood from a phylogenetic perspective. The homogeneity found both within and among the groups of repeats, as mentioned above, is the result of concerted evolution, which means that, the closer the species are in genetic terms, the more similar their repetitive motif sequences are (Ellingsen et al., 2007; Schirrmeister et al., 2012; De Luca et al., 2021; Zhang et al., 2021). This, together with their arrangement in the tree, implies that these types of repeats share a common ancestry (Belyayev et al., 2019; Jiang et al., 2024).

However, interestingly, some species lack repeats, suggesting that repeats may have been lost during the evolutionary process from an ancestor in which this feature was present. For example, *Epinephelus latifasciatus* and *Epinephelus aeneus* are the only species in their clade that do not have an *in tandem* repeats (Table 1; Figure 1). This suggests that the absence of repeats in some species is likely due to random loss through genetic drift (Xiao et al., 2017; Butenko et al., 2024) or, possibly, differential selection pressures. 

Several studies have reported that sequence features associated with replication and transcription control can be conserved within mitochondrial genomes (Fernández-Silva et al., 2003; San Mauro et al., 2006; Zhuang et al., 2013; Xu et al., 2024). This may help explain, at least in part, the maintenance of certain repeats, given that the mitochondrial CR functions as the origin of H-strand replication and contains promoters involved in transcription from both the light and heavy strands (Kurabayashi and Sumida, 2013; Zhuang *et al*., 2013). In addition, expansions or contractions of the full repeat region can change the local sequence context and spacing near the CR boundaries. In that sense, such changes may be associated with variation in adjacent regions, including nearby coding genes, through shifts in genome organization rather than by directly affecting coding function (Zardoya et al., 1995; Dadkhah et al., 2023).

Consistent with this interpretation, repeat-driven changes in CR architecture have also been linked to length variation in the mitochondrial CR across diverse taxa. For example, Liu et al. (2013) demonstrated that the mitochondrial genomes of the clam *Scapharca broughtonii* ranged in length from approximately 47 kb to approximately 50kb due to the variation in the number of repeat copies. In this case, the number of tandem repeats found in this segment is correlated with the size of the CR. Lin et al. (2021) also reported tandem repeat motifs rich in GCs in the CR of the parasitoid wasp *Nasonia vitripennis* and concluded that these repeats are responsible for the expansion of the size of this region. This is because the repeat motifs found in this species may be folded into large palindromic structures, similar to loops, which contribute to the expansion of these motifs and, in turn, the size of the CR. 

A strong relationship between motif length and the number of times these units were repeated was also observed in the *Epinephelus* species analyzed in the present study (Table 1 and Table S2). This finding is corroborated and supported by the correlation test used in our study, which revealed that motif length is strongly and inversely associated with repeat copy number. This is a key result for the biological question we raised, because it directly supports the hypothesis that tandem repeat architecture is shaped by a trade-off between motif length and repeat copy number within a constrained array.

Moreover, CR length was strongly positively associated with overall mitogenome length (Table S2). In addition, mitogenome length increased with the total size of the full repeat region within the CR (Table S2), suggesting that repeat-rich segments can contribute, at least in part, to broader variation in mitogenome length. However, the most direct signal remains within the CR itself. In this case, the mitochondrial CR length increased with the length of the full repeat region. In other words, because the full tandem repeat region occupies a finite portion of the CR, variation in how much sequence it spans translates into measurable differences in mitochondrial CR length (Wang et al., 2015; Zhao et al., 2015).

Our data do not support the view that repeat variation alone explains mitochondrial CR or mitogenome length. Instead, repeats appear to represent one component within a broader set of factors shaping length variation (e.g., variation in intergenic spacers, localized insertions or deletions, and other lineage-specific structural features), and their relative contribution is likely to differ among lineages and/or species. Even so, the strong associations observed here, both between CR length and full repeat region length and between mitogenome length and repeat region length, indicate that repeats can represent a measurable component of length variation in this group.

These findings reinforce the notion that tandem repeat architecture contributes to length variation in the mitochondrial CR, and they are compatible with the idea that the CR of these organisms may be under constraints that limit its overall size (Hassanin et al., 2009).

### Concerted evolution and implications of *tandem* repeats for the phylogeny of Epinephelidae species

**Figure 2 -:**
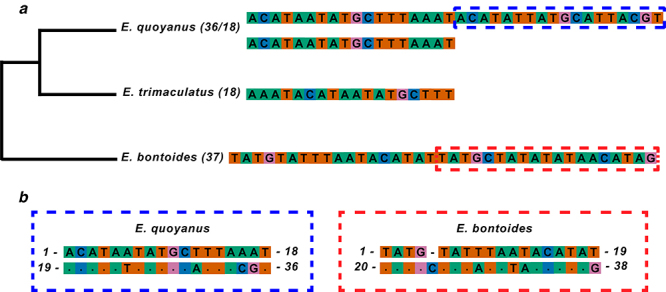
(**a**) Phylogenetic relationships among *E. quoyanus*, *E. trimaculatus*, and *E. bontoides*. The tandem repeats found in these species are shown beside them. The dotted rectangles indicate the sequences highlighted in (**b**). In *E. quoyanus*, the same repeat may have two distinct forms (18 and 36 bps). The alignment of the halves of the sequences of 36 bps and 38 bps in *E. quoyanus* and *E. bontoide* (**b**) reveals some differences, which are clearly the result of mutations in a single ancestral repeat.

While the short and medium repeats are distributed throughout the phylogenetic tree, the long repeats are limited to a specific monophyletic clade (Figure 1). Short repeats are found all over the phylogenetic tree, and sometimes, closely related species (sister groups) can share the same basic repeat motif sequence, such as *E. moara* and *E. bruneus*, which have the same repeat size (17 bps) within the same region (between bases 1-372/373). Other groups possess different sizes of repeats, such as the monophyletic group composed of *E. quoyanus* (repeat motif with 18 or 36 bps), *E. trimaculatus* (18 bps) and *E. bontoides* (37 bps). These kinds of groups could be the keys to understanding how the repeat size evolves over time (Figure 2 a)*.* Here, we observed a clear alignment between the first 18 bases of the *E. quoyanus* repeat sequence and the 18 latter bases (Figure 2 b ), with only four differences. The same phenomenon was observed in the *E. bontoides* repeat sequence alignment (Figure 2 b ).

As noted above, with respect to the repeats of *E. quoyanus*, the analysis revealed two types of repeats in the same region (Figures S1a and S1b). This reflects a limitation of Tandem Repeats Finder analysis due to the characteristics observed in the repeat motif length of this region, but in fact, *E. quoyanus* has only a single type of repeat size. Initially, a more systematic analysis revealed that the 18 bps repeats of *E. quoyanus* are not homogeneous, but rather, are repeated in a nonconsecutive manner (Figure S1a), whereas the 36 bps motif is repeated regularly and conserved *in tandem* (Figure S1b). Analysis of the short motifs revealed that odd repeats are homogeneous but differ in four bases from even repeats, which are also homogeneous (Figure 2 b  and S1a).

**Figure 3 -:**
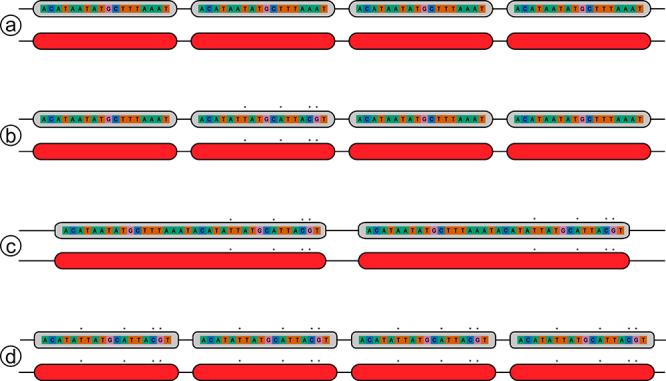
Effects of in-concert evolution on the Control Region: (**a**) the core of a repeat, repeated four times; (**b**) four hypothetical mutations (*) in the second repeat; (**c**) homogenization derived from the in-concert evolution of a core made up of repeats 1 and 2, with a mean repeat of 36 bps; (**d**) potential competition between the short (ancestral) and medium (derived) repeats, resulting in homogenization of the short repeat, which is now different from the ancestral short repeat (**a**).

In other words, in the case of *E. quoyanus*, this appears to be a repeat of 36 bps (Figure 3 a ) that has been changed by mutations (Figure 3 b ) and the concerted homogenization of a motif that now contains two different repeat motif lengths in one, one odd and one even (Figure 3 b-c ). That is, it can be recognized as a single repeat motif length, now of 36 bps (Figures 2 a  and 3c).

If this is true, we could expect that other species of the same clade, with similar motif length patterns, would show the same pattern, that is, an increase in the size of the motif. First, the repeat of *E. bontoides* has only a single repeat motif sequence of 37 bps, which is highly similar to the 36 bps repeat sequence observed in *E. quoyanus* (Figure 2 a )*.* Second, the comparison of the 5’ (16 bps) and 3’ (17 bps) regions of the repeat of *E. bontoides* (Figure 2 b) revealed a similar pattern to that observed in *E. quoyanus* (Figures S1c and S1d), with five differences in the bases between the two regions. In other words, both species present the same pattern of motif expansion, with the motifs of the different repeats in both species being formed by random mutations and concerted evolution independently.

This pattern is also observed in *E. trimaculatus*, which has a short repeat of 18 bps (Figure 3 a ), likely reflecting a subsequent reduction in size. This is possible because the different sizes of the repeat motifs compete to occupy the 5’ region of the CR and may become shorter again through a new round of concerted evolution (Figure 3 d ).

One last example supports the hypothesis of the growth of the repeat motif. As mentioned above, two repeats of different sizes are found *in tandem* in *E. chlorostigma* (Table 1)*.* A comparison of the two motifs revealed that the medium (76 bps) and short (24 bps) repeats can be easily aligned, as observed in the species mentioned above (Figure 2). However, this medium repeat is not an exact copy of three different motifs of ancestral tandem repeats, which may reflect the long period of time since the medium repeat was formed. This would increase the chance of new mutations and increase the motif length, which would ultimately result in greater differentiation within the repeat. In any case, these two repeats are arranged side by side and represent a clear example of sequences that compete to accumulate the largest possible number of copies.

This means that these sequences behave selfishly and, by genetic drift, may be able to increase either their frequency or size without necessarily being advantageous or disadvantageous in terms of the fitness of the organism while also guaranteeing their transmission (Hurst and Werren, 2001; Gardner and Úbeda, 2017; McLaughlin and Malik, 2017). A similar pattern is found in many groups of animals; in the present case, there is a selfish dispute among sequences with different motif lengths, which can ensure their dispersal and fixation (Klucnika and Ma 2019; Dubie et al., 2020; Wagner et al., 2020; Butenko et al., 2024; Sequeira et al., 2024).

### Supplementary Material

Figure S1 - Results of the analysis run at Tandem Repeats Finder.

Table S1 - Species of the Epinephelidae family included in the phylogenetic reconstruction.

Table S2 - Correlation analyses of the relationships between tandem repeat architecture and sequence length metrics in Epinephelus mitochondrial control regions.

## Data Availability

All the data used to support the findings of this study are included within the article.
