# Supplementary material for: Comparative synthesis of tandem repeats in the control region of Epinephelus mitogenomes (Peciformes: Epinephelidae)
Source: Genet Mol Biol. 2026 May 15;49(Suppl 1):e20250240. doi: 10.1590/1678-4685-GMB-2025-0240 (PMC13227324; doi:10.1590/1678-4685-GMB-2025-0240)
Supplement: Figure S1 - [file 1415-4757-GMB-49-s1-e20250240-s1.pdf]

Genetics and Molecular Biology
https://doi.org/10.1590/1678-4685-GMB-2025-0240

Supplementary Material to "Comparative synthesis of tandem repeats in the control region of Epinephelus mitogenomes (Peciformes: Epinephelidae)"

Figure S1 - Results of the analysis run at Tandem Repeats Finder. Two different results were found for *E. quoyanus*, a repeat of 18 bps (a) and a repeat of 36 bps (b). In each comparison, the upper values are the number of bases in the mitochondrial Control Region of the respective species (i.e. 181 or 173), whereas the lower values are the repeats identified in the species by TRF (i.e. 1). The asterisks (*) indicate the differences between the core of the repeat and the sequence of the Control Region. In (b), the dotted rectangles outline the odd pairs of repeats identified in (a). The results for *E. bontoides* are shown in (c), while the sequences inside and outside the rectangle are aligned in (d). Once again, the asterisks (*) indicate differences between the comparisons.

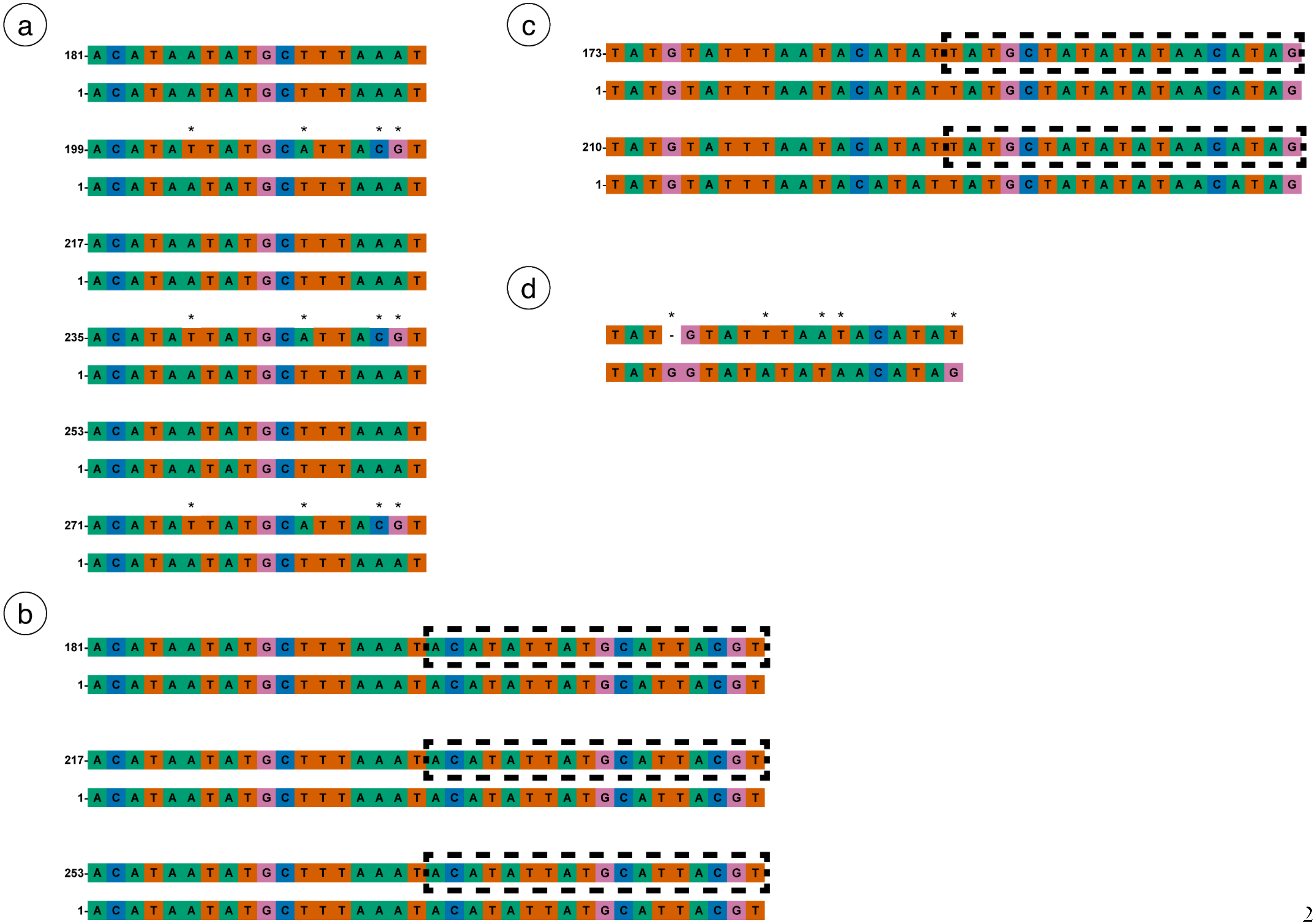
a
181- A C A T A A T A T G C T T T A A A T
1- A C A T A A T A T G C T T T A A A T
199- A C A T A T T A T G C A T T A C G T
1- A C A T A A T A T G C T T T A A A T
217- A C A T A A T A T G C T T T A A A T
1- A C A T A A T A T G C T T T A A A T
235- A C A T A T T A T G C A T T A C G T
1- A C A T A A T A T G C T T T A A A T
253- A C A T A A T A T G C T T T A A A T
1- A C A T A A T A T G C T T T A A A T
271- A C A T A T T A T G C A T T A C G T
1- A C A T A A T A T G C T T T A A A T
b
181- A C A T A A T A T G C T T T A A A T A C A T A T T A T G C A T T A C G T
1- A C A T A A T A T G C T T T A A A T A C A T A T T A T G C A T T A C G T
217- A C A T A A T A T G C T T T A A A T A C A T A T T A T G C A T T A C G T
1- A C A T A A T A T G C T T T A A A T A C A T A T T A T G C A T T A C G T
253- A C A T A A T A T G C T T T A A A T A C A T A T T A T G C A T T A C G T
1- A C A T A A T A T G C T T T A A A T A C A T A T T A T G C A T T A C G T
c
173- T A T G T A T T T A A T A C A T A T T A T G C T A T A T A T A A C A T A G
1- T A T G T A T T T A A T A C A T A T T A T G C T A T A T A T A A C A T A G
210- T A T G T A T T T A A T A C A T A T T A T G C T A T A T A T A A C A T A G
1- T A T G T A T T T A A T A C A T A T T A T G C T A T A T A T A A C A T A G
d
T A T - G T A T T T A A T A C A T A T
T A T G G T A T A T A T A A C A T A G
