# Supplementary material for: Comparative synthesis of tandem repeats in the control region of Epinephelus mitogenomes (Peciformes: Epinephelidae)
Source: Genet Mol Biol. 2026 May 15;49(Suppl 1):e20250240. doi: 10.1590/1678-4685-GMB-2025-0240 (PMC13227324; doi:10.1590/1678-4685-GMB-2025-0240)
Supplement: Table S1 - [file 1415-4757-GMB-49-s1-e20250240-s2.pdf]

Genetics and Molecular Biology
https://doi.org/10.1590/1678-4685-GMB-2025-0240

Supplementary Material to "Comparative synthesis of tandem repeats in the control region of Epinephelus mitogenomes (Peciformes: Epinephelidae)"

Table S2 - Correlation analyses testing relationships between tandem repeat architecture and sequence length metrics in *Epinephelus* mitochondrial CR. Both Spearman's ρ and Pearson's r are reported, with two sided p values; sample size (n) corresponds to the number of records with complete data for each comparison. Italic correspond to significant values. Mitogenomes presenting more than one distinct tandem repeat array in the CR were excluded from these analyses to avoid conflating repeat architectures within a single record.

Test	Relationship tested	n	Spearman's ρ	*P-value*	Pearson's r	*P-value*
1	CR length vs Mitogenome length	50	-	-	0,7565	*2,1E-10*
2	Mitogenome length vs Full repeat region length	50	0,461753	*0,000737*	-	-
3	CR length vs Full repeat region length	50	0,74568	*5,2E-10*	-	-
4	Motif length vs Repeat copy number	50	-0,76358	*1,13E-10*	-	-
